# Simultaneous Immunization against Tuberculosis

**DOI:** 10.1371/journal.pone.0027477

**Published:** 2011-11-16

**Authors:** Elma Z. Tchilian, Edward O. Ronan, Catherine de Lara, Lian Ni Lee, Kees L. M. C. Franken, Martin H. Vordermeier, Tom H. M. Ottenhoff, Peter C. L. Beverley

**Affiliations:** 1 The Peter Medawar Building for Pathogen Research, University of Oxford, Oxford, United Kingdom; 2 Department of Infectious Diseases, Leiden University Medical Centre, Leiden, The Netherlands; 3 TB Immunology Group, The Veterinary Laboratories Agency, Weybridge, United Kingdom; Fundació Institut d'Investigació en Ciències de la Salut Germans Trias i Pujol, Universitat Autònoma de Barcelona, CIBERES, Spain

## Abstract

**Background:**

BCG, the only licensed vaccine against tuberculosis, provides some protection against disseminated disease in infants but has little effect on prevention of adult pulmonary disease. Newer parenteral immunization prime boost regimes may provide improved protection in experimental animal models but are unproven in man so that there remains a need for new and improved immunization strategies.

**Methods and Findings:**

Mice were immunized parenterally, intranasally or simultaneously by both routes with BCG or recombinant mycobacterial antigens plus appropriate adjuvants. They were challenged with *Mycobacterium tuberculosis* (*Mtb*) and the kinetics of *Mtb* growth in the lungs measured. We show that simultaneous immunization (SIM) of mice by the intranasal and parenteral routes is highly effective in increasing protection over parenteral BCG administration alone. Intranasal immunization induces local pulmonary immunity capable of inhibiting the growth of *Mtb* in the early phase (the first week) of infection, while parenteral immunization has a later effect on *Mtb* growth. Importantly, these two effects are additive and do not depend on priming and boosting the immune response. The best SIM regimes reduce lung *Mtb* load by up to 2 logs more than BCG given by either route alone.

**Conclusions:**

These data establish SIM as a novel and highly effective immunization strategy for *Mtb* that could be carried out at a single clinic visit. The efficacy of SIM does not depend on priming and boosting an immune response, but SIM is complementary to prime boost strategies and might be combined with them.

## Introduction

Development of effective booster vaccines is hampered by lack of understanding of protective immunity to tuberculosis or BCG-induced protection [1]. In animals, parenteral immunization with recombinant (rec) viruses containing *Mtb* genes generates powerful immune responses, but has only weak or transient protective effects. Nor have these vectors generally boosted protection effectively when administered parenterally after BCG priming [2]–[8]. Several adjuvanted rec *Mtb* proteins have shown protection equal to BCG; however, after BCG priming the increase in protection afforded by parenteral boosters is variable. The most effective regimes require repeated administration of rec *Mtb* fusion proteins [9]–, sometimes combined with further booster doses of BCG [12]. In contrast, immunization via the respiratory tract is frequently highly effective: both rec proteins and viral vectors induce protective immunity and boost parenteral BCG-induced protection [5], [13]–[16]. Respiratory administration of spray dried BCG is highly effective in guinea pigs [17].

Following aerosol infection of mice with *Mtb*, activated antigen specific T cells are not detected in the mediastinal lymph nodes until 9 days and in the lungs until 14 days post infection. During this early phase of infection the mycobacterial load increases logarithmically, but after the first few weeks the primary response to mycobacteria partially contains mycobacterial growth, so that the mycobacterial load stabilizes and increases much more slowly or not at all [18], [19]. Remarkably, parenteral immunization with BCG, *Mtb* or subunit vaccines only slightly accelerates the immune response, so that when mice are challenged with *Mtb* the kinetics of pulmonary *Mtb* growth do not differ between naïve and immune mice for the first 14 days [2], [20], [21], although after the initial phase of logarithmic growth the mycobacterial load stabilizes at a lower level than in naïve mice. Thus it appears that systemic immunity only begins to inhibit *Mtb* growth relatively late after pulmonary infection. In contrast, protective immunity following intranasal (i.n.) immunization of mice with a rec adenovirus expressing *Mtb* antigen 85A (Ad85A) induces a large pulmonary population of activated, dividing CD8 effector T cells, many of which can be recovered by broncho-alveolar lavage (BAL) [2], [22]. *Mtb* growth in Ad85A i.n. immunized mice is inhibited during the first week after infection but the mycobacteria then grow logarithmically before the mycobacterial load again stabilizes at a lower level than in naïve mice [2]. Stabilization may be due both to a primary response to mycobacterial antigens absent from the vaccine, as well as recruitment of cells induced in the systemic immune compartment by i.n. immunization. Nevertheless, these data suggest that combining i.n. and parenteral immunization might be effective because i.n. immunization generates a pulmonary immune response that can inhibit *Mtb* growth early after infection, while parenteral immunity can further inhibit growth later. If this is the case, simultaneous immunization (SIM) with pulmonary and parenteral vaccines might be as effective as priming and boosting by these two routes and would have the advantage of requiring only one simultaneous immunization procedure. In this study we set out to test whether SIM by the parenteral and intranasal routes in mice would provide improved protection over the “gold standard” immunization procedure of parenteral BCG. Here we report that SIM, which harnesses both local pulmonary and systemic immunity, induces much more powerful protective immunity than BCG alone and indeed does not depend on priming and boosting.

## Methods

### Ethics

All animal work was carried out in accordance with the UK Animal (Scientific Procedures) Act 1986 and was approved by the animal use ethical committee of Oxford University.

### Animals and Immunization

**Figure 1:**
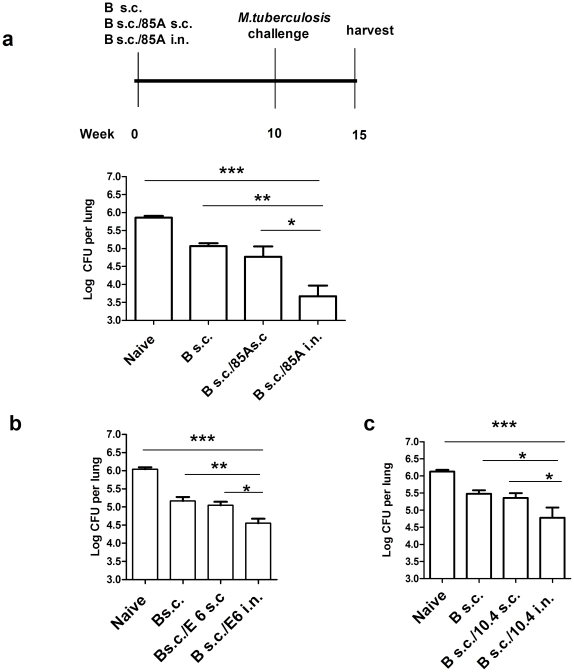
*Mtb* cfu after SIM with BCG and subunit vaccines. **a.** C57BL/6 mice were immunized once with BCG with or without simultaneous administration of 85A s.c. or 85A i.n., or **b,** once with BCG with or without simultaneous administration of E6 s.c. or E6 i.n. and **c,** once with BCG with or without simultaneous administration of 10.4 s.c. or 10.4 i.n.. Ten weeks after the last immunization they were challenged with *Mtb* i.n. and a further 5 weeks sacrificed later for enumeration of lung *Mtb* cfu. Representative data from one of two experiments with 5–7 mice/group are shown. ***p<0.001, **p<0.01, * p<0.05 between the indicated groups, one-way ANOVA with Tukey's post test. Data are means ± s.e.m.

**Figure 2:**
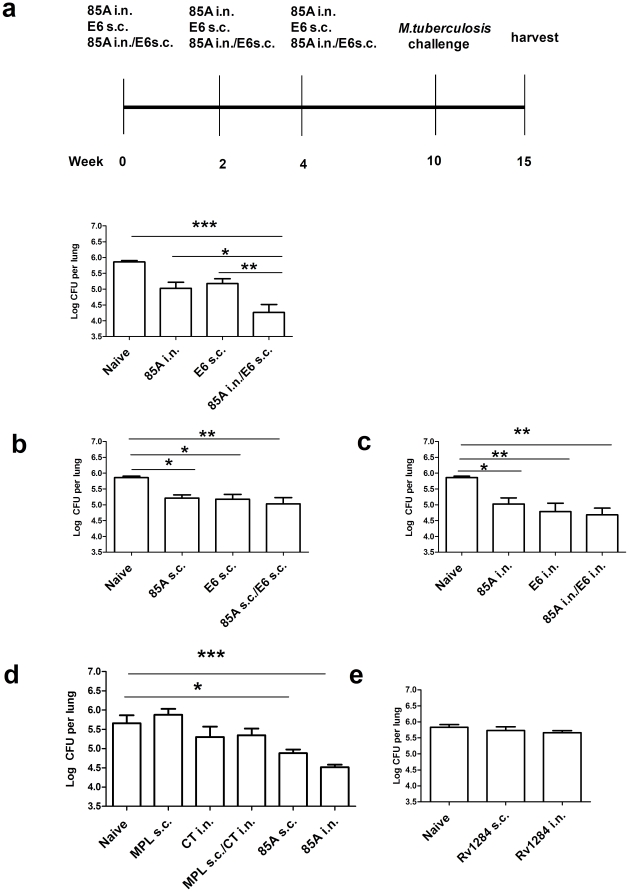
*Mtb* cfu after SIM with subunit vaccines. **a.** C57BL/6 mice were immunized 3 times at 2 weekly intervals with 85A s.c., or E6 i.n. either alone or in combination with appropriate adjuvants. Six weeks after the last immunization mice were challenged with *Mtb* and sacrificed for lung cfu enumeration 5 weeks later. **b.** Mice were immunized 3 times with 85A s.c. or E6 s.c. with MPL, separately or in combination and then challenged. **c,** the same antigens were administered 3 times i.n. with CT before challenge. **d.** Mice were immunized with 85A i.n. or s.c. in appropriate adjuvants or MPL or CT were administered 3 times s.c. or i.n separately or simultaneously and **e,** Rv1284 was administered s.c. or i.n. 3 times with appropriate adjuvants before *Mtb* challenge and enumeration as in **a**. Representative data from one of two experiments with 5–7 mice/group are shown. ***p<0.001, **p<0.01, * p<0.05 between the indicated groups, one-way ANOVA with Tukey's post test. Data are means ± s.e.m.

**Figure 3:**
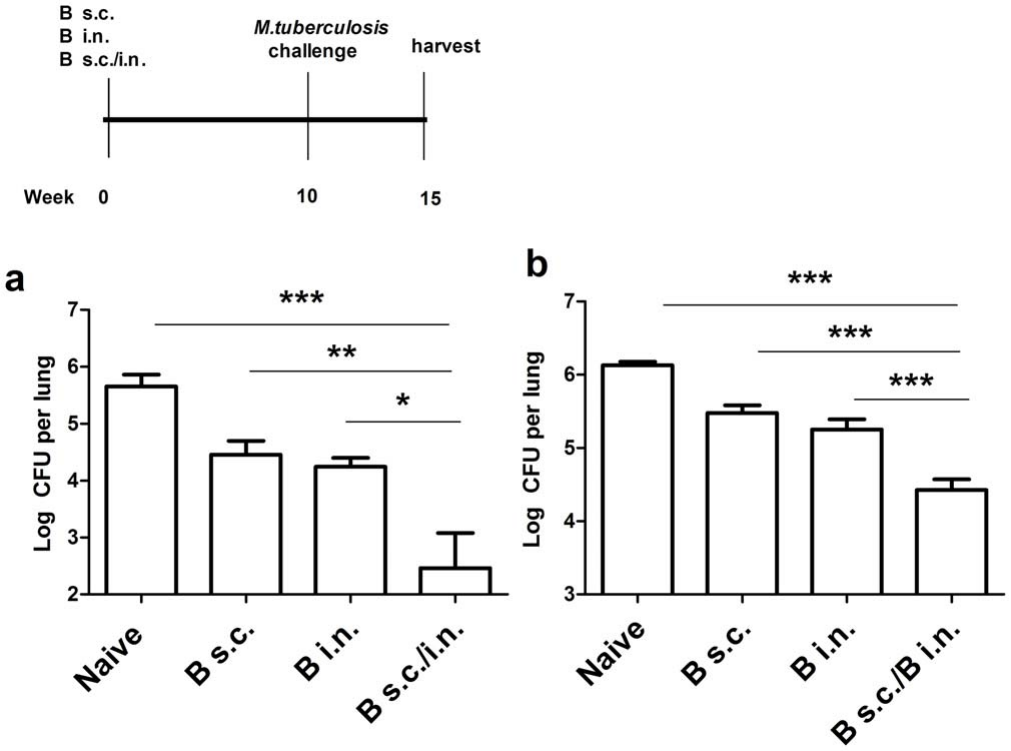
*Mtb* cfu after SIM with BCG. **a** and **b.** In two experiments, C57BL/6 mice were immunized once with BCG s.c. or BCG i.n. or simultaneously with the same dose of BCG divided between the s.c./i.n. routes. Ten weeks later they were challenged with *Mtb* and lung cfu enumerated 5 weeks later. Data from the two experiments with 5–7 mice/group are shown. ***p<0.001, **p<0.01, * p<0.05 between the indicated groups, one-way ANOVA with Tukey's post test. Data are means ± s.e.m.

All experiments were performed with 6–8 week old female C57BL/6 mice (Harlan Orlac, Blackthorn, UK). Mice were immunized with 2×10^5^ colony forming units (cfu) BCG (SSI, Copenhagen, Denmark) s.c. on the flank in 200 µl PBS. For i.n. immunization, mice were anesthetized with isoflurane and 2×10^5^ cfu BCG in 40 µl PBS was administered with a pipette, divided between the two nostrils. For SIM with BCG 1×10^5^ cfu were administered s.c. and 1×10^5^ cfu i.n. Mice were also immunized with 4 µg rec antigen 85A protein (85A), prepared as described previously [23], 20 µg of a synthetic peptide encoding the first 20 amino acids of the 6 kDa early secreted antigenic target (E6) (Peptide Protein Research Ltd, Fareham, UK), 4 µg rec antigen TB10.4 (Proteix, Prague, Czech Republic) or 4 µg of the enduring hypoxia response protein Rv1284 [24]. Rv1284 was sub-cloned into the expression vector pET104-DEST42 (Invitrogen, Paisley, UK) from a complete Gateway Clone set from *Mtb* obtained through NIAID's Pathogen Functional Genomics Resource Center, managed and funded by Division of Microbiology and Infectious Diseases, NIAID, NIH, DHHS and operated by the J. Craig Venter Institute. After expression in BL21(DE3) *E. coli* cells (Invitrogen) as a His-tagged protein it was purified using His SpinTrap columns (GE Healthcare, Chalfont St Giles, UK). Subcutaneous (s.c.) immunization was performed by injecting each of the antigens, half subcutaneously and half intra-muscularly in 200 µl monophosphoryl lipid A Sigma adjuvant system (MPL) (Sigma, Poole, UK) prepared according to the manufacturer's instructions. For i.n. protein immunization, mice were anesthetized with isoflurane and the same doses of the antigens mixed with 2 µg of cholera toxin (CT) (Sigma) were pipetted into the nostrils in a total volume of 40 µl. In most experiments, proteins were administered 3 times at 2 weekly intervals as indicated in the figure legends. Mice were also immunized once at the same time with BCG s.c. and either 4 µg 85A, 20 µg E6 or 4 µg TB10.4. The subunit vaccine was administered either s.c. with MPL or i.n. with CT as described above. Three types of experiment were performed. 1) BCG was administered s.c. alone or administered simultaneously with a recombinant protein or synthetic peptide antigen s.c. with MPL or i.n. with CT. The mice were challenged 10 weeks later with *Mtb* (see Fig. 1 and 2) Subunit antigens were administered three times s.c., i.n. or simultaneously, with MPL or CT as appropriate, by the two routes at two weekly intervals. Adjuvant only controls were included in some experiments. Six weeks after the last immunization, the mice were challenged with *Mtb* (see Fig. 2 and 3) BCG only was administered without adjuvant, s.c., i.n. or simultaneously by both routes (Fig. 3). The mice were challenged 10 weeks later.

### Isolation of lung and spleen lymphocytes

Lungs were perfused with PBS, cut into small pieces and digested with 0.7 mg/ml collagenase type I (Sigma) and 30 µg/ml DNase I (Sigma) for 45 min at 37°C. Lung fragments were then crushed through a cell strainer using a 5 ml syringe plunger, washed, layered over Lympholyte (Cederlane, Ontario, Canada) and centrifuged at 1000×g for 25 min. Interface cells were collected and washed. Spleens were passed through a cell strainer using a 5 ml syringe plunger, red blood cells were lysed using RBC lysis buffer (Qiagen, Crawley, UK) and the cells were washed.

### Flow cytometry

Cells were cultured in Hepes buffered RPMI supplemented with 10% heat-inactivated FCS, L-glutamine, penicillin and streptomycin for 6 hours. Cells were stimulated with *Mtb* purified protein derivative (PPD) at 10 µg/ml (SSI) for 12 hours, or a pool of 66 15mer peptides overlapping by 10 amino acids and covering the entire sequence of 85A or E6 20mer (Peptide Protein Research Ltd) for 6 hours. Each peptide was at a final concentration of 2 µg/ml during the stimulation. After 6 (for PPD) and 2 (for the peptide pool) hours at 37°C, Golgi Plug (BD Biosciences, Oxford, UK) was added according to the manufacturer's instructions before intracellular cytokine staining.

Cells were washed and incubated with CD16/CD32 monoclonal antibody to block Fc binding. Subsequently the cells were stained for CD4 (RM4-5), CD8 (53-6.7) (BD Bioscience, Oxford, UK) IFNγ (XMG1.2), IL-2 (JES6-5H4) and TNF (MP6-XT22) (eBioscience, Hatfield, UK) using the BD Cytofix/Cytoperm kit according to the manufacturer's instructions. Cells were fixed with PBS+1% paraformaldehyde, run on a LSRII (BD Biosciences) and analyzed using FlowJo software (Tree Star Inc, Ashland, Oregon, USA). Three or 4 mice from each experimental group were used for immunological analysis.

### Infection with *Mtb* and determination of mycobacterial load

Five to 7 mice were anesthetized with isoflurane and infected i.n. with *Mtb* (Erdman strain, kindly provided by Dr. Amy Yang, CBER/FDA) in 40 µl PBS. Lung cfu were enumerated 24 hours after challenge to determine the number of organisms deposited, which was of the order of ∼200 cfu. Mice were sacrificed at indicated times, the lungs were homogenized and the lung mycobacterial load determined by plating 10-fold serial dilutions of tissue homogenates on Middlebrook 7H11 agar plates (E&O Laboratories Ltd, Bonnybridge, UK). Colonies were counted after 3–4 weeks of incubation at 37°C in 5% CO_2_.

### Statistical Analysis

All results are representative of at least 2 independent experiments with similar results. Data were analyzed using one-way ANOVA followed by Tukey's multiple comparison test.

## Results

### SIM with BCG and subunit vaccines

We first tested the efficacy of SIM by administering BCG subcutaneously (s.c.) and recombinant *Mtb* antigen 85A protein (85A) i.n. with cholera toxin (CT) as a model mucosal adjuvant. In this experiment 85A with CT was given only once at the same time as BCG s.c. so that conventional priming and boosting was not possible. SIM animals were compared to naive or BCG s.c. controls or mice given both BCG and 85A s.c. with monophosporyl lipid A (MPL) as adjuvant (Fig. 1a). As expected, BCG s.c. suppressed *Mtb* growth by 0.8 log_10_ compared to naive animals. However, SIM with BCG s.c./85A i.n., targeting both pulmonary and systemic immunity, provided strikingly increased protection, reducing *Mtb* colony forming units (cfu) by an additional 1.3 log_10_ compared to BCG s.c. alone. BCG s.c./85A s.c., targeting only systemic immunity, did not differ from BCG alone (Fig. 1a). To confirm the generality of this effect we carried out SIM with two other *Mtb* antigens, a synthetic peptide encoding the first 20 amino acids from the 6 kDa early secretory antigenic target ESAT6 (E6) and rec protein TB10.4. Both BCG s.c./E6 i.n. or BCG s.c./10.4 i.n. induced significant additional decreases in pulmonary *Mtb* load (by 0.6 log_10_) compared to BCG alone), while BCG s.c./E6 s.c. and BCG s.c./10.4 s.c. did not (Figs. 1b and c).

### SIM with two subunit vaccines

Since SIM with BCG s.c./E6 i.n. provided additional protection over BCG s.c. alone and BCG does not contain E6, this experiment strongly suggested that SIM does not require priming and boosting. To establish this more definitively, we used two non cross-reactive antigens, 85A and E6. Since adjuvanted rec protein vaccines are generally administered repeatedly [9], [10], we gave the 2 subunit vaccines together or separately with appropriate adjuvants by the parenteral or pulmonary routes three times at 2 weekly intervals. The mice were challenged with *Mtb* 6 weeks after the last immunization (Fig. 2a). SIM with 85A and E6, targeting pulmonary and systemic immunity, decreased the mycobacterial load by 1.6 log_10_ compared to naïve mice. Immunization with one antigen by either the pulmonary or parenteral route alone had a lesser effect (Fig. 2a) and when both antigens were given together either s.c. or i.n., there was no significant increase compared to one subunit alone (Fig. 2b and c). In one experiment (not shown), both 85A i.n./E6 s.c. (as in 2A) and 85A i.n./E6 i.n. (as in 2C) were included. In this experiment, there was no significant difference between these two groups but only the SIM group (85A i.n./E6 s.c.) differed significantly from the single antigen controls, implying that SIM is more effective than two antigens i.n.

These subunit vaccine experiments show that the increased protection of SIM is not due to a prime boost effect. However, because adjuvant effects are important in immunity to *Mtb* [12], [25], we also immunized mice 3 times with 85A i.n.+CT, 85A s.c.+MPL or with CT i.n., MPL s.c. or both simultaneously (Fig. 2d). The mycobacterial burden in the adjuvant controls did not differ from naïve animals even when calculated by T test (naïve v CT p = 0.33, naïve v MPL p = 0.41 and naïve v CT/MPL p = 0.29), although 85A given i.n. or s.c. with appropriate adjuvants provided significant protection. Additionally we immunized mice 3 times with the hypoxia induced rec protein Rv1284 [24] s.c. or i.n. with CT or MPL. Rv1284 immunized mice (Fig. 2e) did not differ from naive animals, indicating that the efficacy of SIM is dependent on induction of antigen specific immune responses in the lungs and systemically. This result provided further evidence for the lack of effect of CT or MPL without an effective antigen. We hypothesize that Rv1284 may be ineffective in C57Bl/6 mice because it lacks H-2^b^ restricted T cell epitopes.

### SIM with BCG/BCG

Finally, since BCG is the gold standard vaccine for animal experiments and the only available human tuberculosis vaccine, we tested SIM with BCG s.c./BCG i.n. This combination was highly effective, reducing the mycobacterial load in two separate experiments by an additional 1.1 and 2.0 log_10_ compared to BCG given by either route alone (1.7 and 3.1 log_10_ compared to naïve mice) (Fig. 3a and b).

### Mechanism of SIM induced additive protection

**Figure 4:**
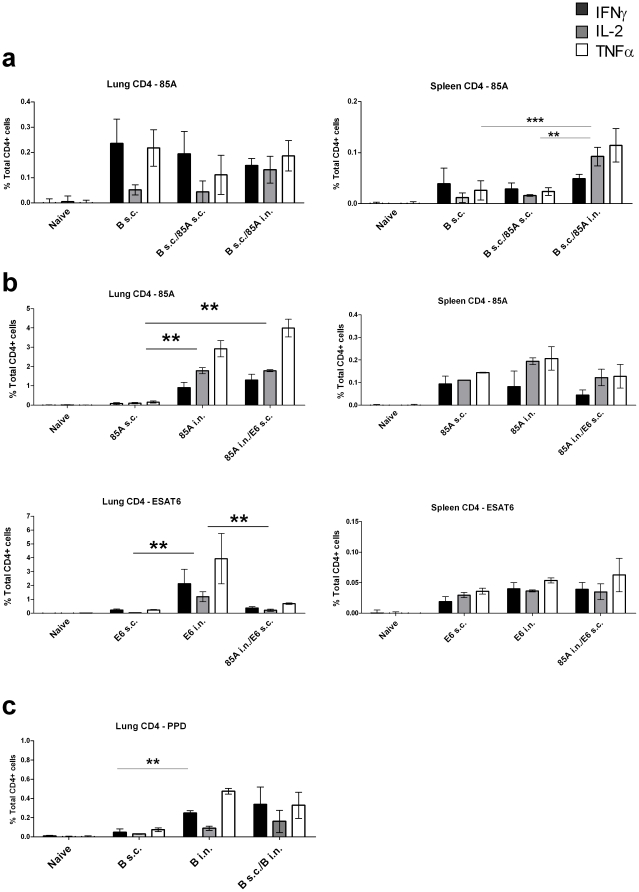
Cytokine responses of lung and spleen T cells to antigens. **a.** Mice were immunized with BCG s.c., BCG s.c./85A s.c. or BCG s.c./85A i.n. as in Fig. 1a. Lung and spleen cells were isolated 10 weeks after immunization and stimulated with pooled 85A peptides for 6 hours. **b.** mice were immunized 3 times at 2 weekly intervals with 85A s.c., 85A i.n., E6 s.c. or E6 i.n. either alone or in combination as in Fig. 2a,b,c and cells isolated 6 weeks after immunization and stimulated with pooled 85A peptides or E6 for 6 hours. **c**, mice were immunized with BCG s.c., BCG i.n. or BCG s.c./i.n. as in Fig. 3b and lung cells were isolated 10 weeks after immunization and stimulated for 12 hours with PPD. After stimulation the proportion of IFNγ, IL-2 and TNF producing cells was determined by flow cytometry of CD4 gated cells (numbers of responding CD8 cells were too low for reliable analysis). Results are expressed as the means ± s.e.m. of 3 or 4 mice per group and are representative of 2 independent experiments. In **a,** ***p<0.001, **p<0.01 indicate significant differences between numbers of spleen IL-2 producing cells only. In **b**,**p<0.01 between all cytokines in the indicated groups and in **c**, **p<0.01 for TNF only. All other groups differ significantly from the naïve group but these comparisons are omitted for simplicity. One-way ANOVA with Tukey's post test.

To investigate the mechanisms underlying the efficacy of SIM, we assayed antigen specific responses in the lungs and spleen at the time of *Mtb* challenge by intra-cytoplasmic cytokine staining (ICS) and flow cytometry [2], [5] for the experiments shown in Figs. 1a, 2a,b,c and 3b. We also analyzed numbers of single or multiple cytokine producing cells in lungs and spleens (data not shown) [5]. In the experiment shown in Figs. 1a and 4a, in which mice were immunized only once with BCG and rec 85A, the responses to 85A in the lungs were low and similar in all groups at 10 weeks post-immunization (Fig. 4a) although the mice immunised with BCG s.c./85A i.n. were very well protected (Fig. 1a). In the experiments shown in Figs. 2a,b,c and 4b, or 3b and 4c, lung responses were higher in i.n. or simultaneously immunized mice. In all experiments the percentages of antigen specific cells in the spleen were lower than in the lungs and overall there were no consistent differences between mice immunized s.c., i.n. or simultaneously. Therefore in these experiments protection did not correlate clearly with the number of antigen specific cells in the lungs or spleen.

**Figure 5:**
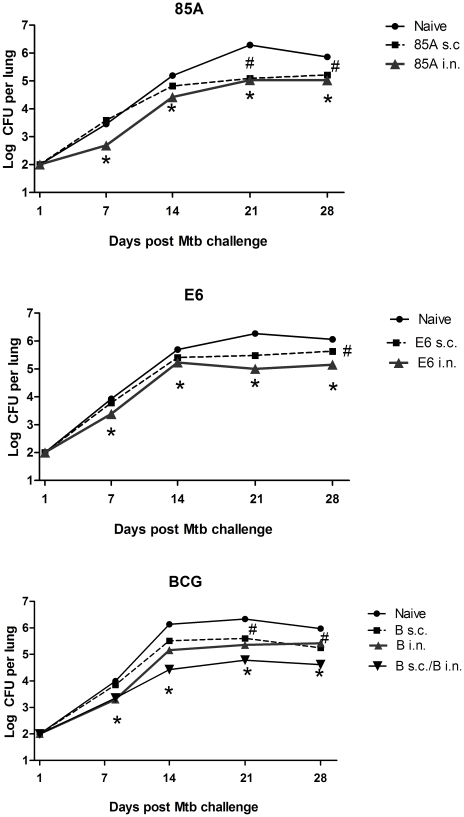
Kinetics of *Mtb* growth after s.c. and i.n. immunization. C57BL/6 mice were immunized once with BCG s.c., BCG i.n. or BCG s.c./BCG i.n. or 3 times at two weekly intervals with 85A s.c., 85A i.n., E6 s.c. or E6 i.n. Ten weeks after immunization with BCG or 4 weeks after the last immunization with 85A or E6, mice were challenged with *Mtb* and groups of 3–5 mice sacrificed 7, 14, 21 and 28 days later for enumeration of lung *Mtb* cfu. **#** indicates a significant difference between s.c immunized and naïve mice and * a significant difference between i.n. or SIM immunized mice and naïve controls, one-way ANOVA with Tukey's post test. Data are means ± s.e.m. Standard errors are small, so that the error bars are within the symbols when not visible.

**Figure 6:**
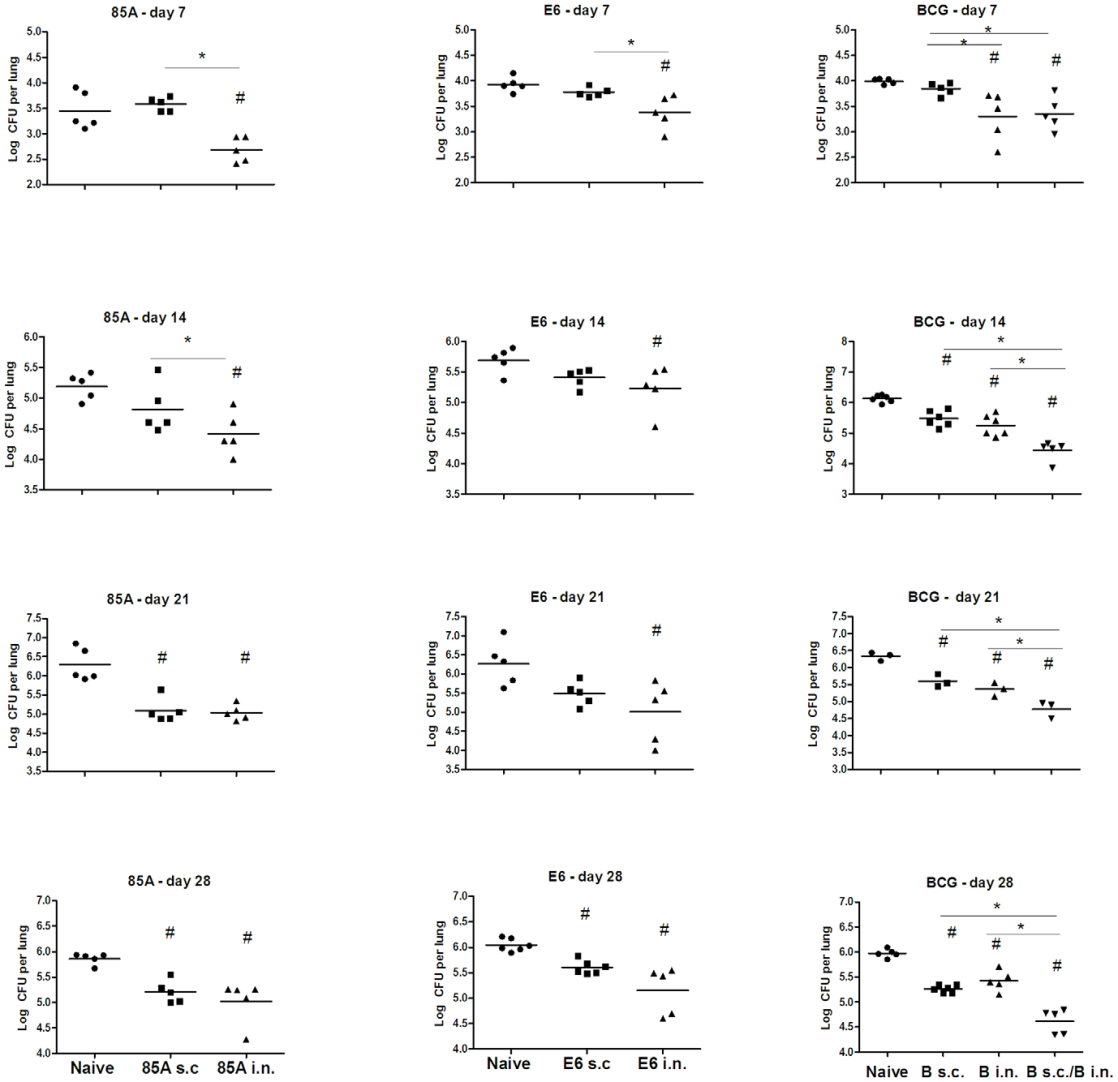
*Mtb* growth in individual mice after s.c. and i.n. immunization. The figure shows results for individual mice from the experiments shown in Fig. 5. C57BL/6 mice were immunized once with BCG s.c., BCG i.n. or BCG s.c./BCG i.n. or 3 times at two weekly intervals with 85A s.c., 85A i.n., E6 s.c. or E6 i.n. Ten weeks after immunization with BCG or 4 weeks after the last immunization with 85A or E6 mice were challenged with *Mtb* and groups of 3–5 mice sacrificed 7, 14, 21 and 28 days later for enumeration of lung *Mtb* cfu. Representative data from one of two experiments are shown. Horizontal lines show group means. **#** indicates a significant difference from naïve mice and * a significant difference between indicated groups. One-way ANOVA with Tukey's post test.

Although we could not discern a definitive correlation between the number and quality of antigen specific cells and protective immunity and both immunization routes induced splenic responses of approximately equal magnitude (Fig. 4), the fact that i.n. but not s.c. immunization always induced populations of antigen specific cells in the lungs, suggested that these may be important for the success of SIM. We therefore turned to *in vivo* analysis of immune function to determine the effect of i.n. or SIM immunization and examined the kinetics of *Mtb* growth after challenge in mice given antigen i.n., s.c. or simultaneously by both routes. All i.n. vaccines that provide additional protection when given simultaneously with parenteral vaccines, inhibited *Mtb* growth by 7 days post-challenge and inhibition compared to naïve mice was also seen at later time points. In contrast, effective parenteral vaccines, including BCG, did not inhibit growth at 7 days but began to do so at 14 days and continued to inhibit at later time points. When the kinetics of mycobacterial growth were examined in SIM mice, both early and additional later inhibition were seen. Figures 5 and 6 show the data from these experiments, either as the kinetics of growth (Fig. 5) or as growth inhibition for individual mice at each time point (Fig. 6).

## Discussion

A distinctive feature of pulmonary infection of mice with *Mtb* is the delay in initiation of a primary immune response in the draining mediastinal lymph nodes, so that *Mtb* grows logarithmically in the lungs during the early phase of infection [18], [19], [26]. Only after the primary response generates effectors that recirculate to the lung does the mycobacterial load stabilize. Even after parenteral immunization with an effective vaccine, BCG, the kinetics of mycobacterial growth are not changed during the first 7 days after challenge, although the mycobacterial load later stabilizes at a lower level than in naïve mice [2]. In contrast, in mice immunized i.n. early *Mtb* growth is inhibited, as well as stabilizing at a lower level than in naïve mice [2]. Intranasal vaccines are often more effective than parenteral immunization against *Mtb* [5], [13], [14], [17] perhaps because i.n. vaccines induce pulmonary immunity that inhibits early mycobacterial growth, while the primary response to non-vaccine antigens may contribute to the later stabilization of mycobacterial load. In any case, we hypothesize that i.n. and parenteral immunization are additive because i.n. immunization induces a pulmonary immune response that inhibits mycobacterial growth early after challenge, while systemic immunity induced by parenteral immunization inhibits mycobacterial growth only later. Because parenteral and pulmonary immunization induce populations of immune cells with differing localization and effects on mycobacterial growth kinetics, we reasoned that their additive effect might not depend on a prime/boost effect and that simultaneous immunization (SIM) should be effective.

The data shown here confirm that BCG s.c. combined with one of three i.n. subunit vaccines, rec 85A, rec TB10.4 or E6 peptide provides increased protection compared to BCG alone. All of the i.n. subunit vaccines or i.n. BCG, inhibit early growth of mycobacteria in the lungs (Figs. 5 and 6). Furthermore, SIM is effective with two non-crossreacting vaccines, rec 85A administered i.n. with cholera toxin as a mucosal adjuvant and ESAT6_1–20_ peptide given parenterally with monophosphoryl lipid A as adjuvant. These experiments demonstrate that prime/boosting is not needed to obtain the additive effect of SIM. Interestingly SIM with BCG s.c./BCG i.n. is also highly effective, a result that contrasts with the finding that boosting BCG primed animals with BCG is generally ineffective. SIM with BCG may be effective not only because it induces early and late inhibition of *Mtb* growth after challenge (Figs. 5 and 6), but also because it circumvents the problem of inhibition of growth of booster BCG in BCG primed animals.

Although inhibition of early and later growth of *Mtb* clearly contribute to the efficacy of SIM, the immune mechanisms require further investigation. Even parenterally administered BCG has been shown to induce changes in expression of lung genes involved in connective tissue responses that last for at least 6 weeks [25] and are important in the host response to *Mtb* [27]. These changes may contribute to the efficacy of SIM when BCG is given s.c. with a second antigen i.n. Furthermore, although we have not seen protective effects of CT and MPL when used without antigens or with Rv1284, the effects of these adjuvants on lung gene expression have not been investigated and may contribute to pulmonary protective immunity. It is also known that the protective effects of BCG against virulent mycobacteria may be lost with time after challenge [28], so that it will be very important in future investigation of SIM, to study both the duration of protective immunity after immunization and the long term maintenance of protection post challenge.

While effects on innate and connective tissue responses may be important for protective immunity to *Mtb*, the adaptive response is clearly also important. Both CD8 T cells induced by Ad85A i.n. [2] or CD4 T cells as shown here and elsewhere [29], can mediate early inhibition of mycobacterial growth. It is also clear that their location within the lung is crucial since although significant numbers of antigen specific cells may sometimes be present in the lungs after parenteral immunization [29], [30], these are found in the lung interstitium, while in contrast i.n. immunization establishes a long-lived population of lung resident, activated, antigen-specific T cells, recoverable by BAL [2], [22], [29]. The BAL population expresses CXCR6 and is highly protective [22], [29]. In the present experiments we did not see a clear correlation between the numbers of antigen-specific cells in the lungs and protective immunity. However we did not study activation-antigen expression on lung lymphocytes, nor did we separate BAL and interstitial lymphocytes. The lack of an observed correlation may therefore indicate that the location within the lung and state of activation of lymphocytes, are more critical than the overall numbers. In future studies of i.n. immunized or SIM mice, it will be important to dissect responses in different lung compartments before drawing conclusions as to the hallmarks of protective i.n. or SIM induced immunity.

SIM with BCG s.c./BCG i.n. is highly effective and sets a new gold standard against which to measure *Mtb* vaccine efficacy (Fig. 3). Another SIM regime, BCG s.c./85A i.n., is highly effective (Fig. 1a) but clearly there are many possibilities for improvement of SIM with subunits, such as the use of multiple antigens (including latency antigens) as fusion genes [11], or recombinant mycobacteria over-expressing immunogenic antigens [1], [31]. Adjuvants and vectors for pulmonary delivery also need to be further developed. SIM has several potential advantages: first, it is highly effective and rapidly establishes a much higher level of protection than BCG alone; second, it only requires immunization procedures that might be performed at a single clinic visit; third, it is compatible with further boosting with subunit vaccines or the employment of recombinant mycobacteria. Some of the latter are as effective as BCG in inducing protective immunity but may be less pathogenic, an important property in HIV+ immuno-compromised individuals [32]. The long-term efficacy, practicality and safety of pulmonary vaccines remain to be thoroughly investigated. However, BCG, rec adenoviruses and rec proteins with adjuvants have all been shown to induce long lasting protection [2], [12]. Cheap, disposable devices to administer spray dried particles or nebulised aerosols for pulmonary vaccination have been developed [33], [34], while respiratory immunization against measles has been shown to be safe and highly efficient. Nebulised BCG has been safely administered to the lungs of >100 children and young adults with no reported ill effects [35].

SIM harnessing both local and systemic immunity is a novel strategy for immunization against *Mtb,* complementary to current parenteral prime boost regimes and with the potential to enhance greatly the efficacy of existing promising subunit vaccines. SIM merits further investigation and development.
